# Identification of drug repurposing candidates based on a miRNA-mediated drug and pathway network for cardiac hypertrophy and acute myocardial infarction

**DOI:** 10.1186/s40246-018-0184-0

**Published:** 2018-12-04

**Authors:** Jiantao Sun, Jiemei Yang, Jing Chi, Xue Ding, Nan Lv

**Affiliations:** 1 Department of Cardiology, the First Affiliated Hospital, Harbin Medical University, Harbin, Heilongjiang People’s Republic of China; 2 Department of Obstetrics, the Second Affiliated Hospital, Harbin Medical University, 246 XueFu Road, Harbin, 150086 Heilongjiang People’s Republic of China

**Keywords:** Drug repurposing, miRNAs, Pathway, Cardiac hypertrophy, Acute myocardial infarction

## Abstract

**Background:**

Cardiac hypertrophy and acute myocardial infarction (AMI) are two common heart diseases worldwide. However, research is needed into the exact pathogenesis and effective treatment strategies for these diseases. Recently, microRNAs (miRNAs) have been suggested to regulate the pathological pathways of heart disease, indicating a potential role in novel treatments.

**Results:**

In our study, we constructed a miRNA-gene-drug network and analyzed its topological features. We also identified some significantly dysregulated miRNA-gene-drug triplets (MGDTs) in cardiac hypertrophy and AMI using a computational method. Then, we characterized the activity score profile features for MGDTs in cardiac hypertrophy and AMI. The functional analyses suggested that the genes in the network held special functions. We extracted an insulin-like growth factor 1 receptor-related subnetwork in cardiac hypertrophy and a vascular endothelial growth factor A-related subnetwork in AMI. Finally, we considered insulin-like growth factor 1 receptor and vascular endothelial growth factor A as two candidate drug targets by utilizing the cardiac hypertrophy and AMI pathways.

**Conclusion:**

These results provide novel insights into the mechanisms and treatment of cardiac hypertrophy and AMI.

## Background

Cardiovascular disease, especially coronary heart disease and stroke, remains the leading cause of death and disability-adjusted life years for all regions worldwide [1]. Cardiac hypertrophy and acute myocardial infarction (AMI) are two types of common cardiovascular diseases. Cardiac hypertrophy is the heart’s response to a variety of extrinsic and intrinsic stimuli that impose increased biomechanical stress [2]. AMI is a common disease with serious consequences in mortality, morbidity, and cost to society [3]. Over the past several years, numerous studies have enhanced our understanding of the mechanism and treatment of cardiovascular health. However, divergent results have created confusion among patients. Drug treatment options and reduction of side effects remain urgent problems for studies of heart disease.

Recent studies have revealed that microRNAs (miRNAs) play essential roles in cardiovascular diseases, including cardiac hypertrophy and AMI. For example, miR-378 suppresses myocardial fibrosis through a paracrine mechanism at the early stage of cardiac hypertrophy following mechanical stress [4]. miR-133 is downregulated in thyroid hormone-mediated cardiac hypertrophy partially via the type 1 angiotensin II receptor [5]. Wei et al. identified miR-101 as an important regulator of cardiac hypertrophy and suggested its potential application in therapy for cardiac hypertrophy [6]. Additionally, many studies have suggested important roles in AMI. For instance, circulating miR-1 and miR-499 have potential as novel biomarkers for AMI [7, 8]. miR-214 inhibits left ventricular remodeling in AMI by suppressing cellular apoptosis via the phosphatase and tensin homolog [9]. In addition, high-throughput microarray and sequencing experiments have identified numerous novel microRNAs in cardiovascular diseases, such as cardiac hypertrophy and AMI [10, 11]. However, many valuable and significant data available for cardiac hypertrophy and AMI had not been abundantly utilized.

Integration of mRNA and miRNA double expression profiles can provide novel insights into research of the mechanisms underlying cardiac hypertrophy and AMI. For example, Yang et al. combined miRNA and mRNA sequencing to identify the protective transcriptome signature of enhanced PI3K signaling in the context of pathological hypertrophy; the results demonstrated that regulation of TGF/miR-21 contributed to the protection of enhanced PI3K signaling against cardiac hypertrophy [12]. Santana et al. compared mRNA and miRNA expression profiles to identify transcriptional and post-transcriptional changes in AMI [13]. Moreover, a network-based method is an effective approach to globally identify biomarkers of disease. For example, Song et al. constructed and analyzed a cardiac hypertrophy-associated long non-coding RNA-mRNA network based on competitive endogenous RNA and revealed functional lncRNAs involved in cardiac hypertrophy. However, these networks have almost exclusively integrated mRNA, miRNA, or lncRNA expression and have lacked drug information.

Research into and development of novel drugs for cardiac hypertrophy and AMI are time-consuming and labor-intensive processes. Recently, drug repurposing has become an essential part of drug discovery that can uncover novel indications for existing drugs [14]. Donner et al. used deep embeddings of gene expression profiles for drug repurposing [15]. Drug repurposing has been successfully used in Parkinson’s disease and cancer [16, 17]. However, few studies have focused on drug repurposing for cardiac hypertrophy and AMI.

In this study, we used heart disease-related genes to construct a miRNA-gene-drug network and analyzed its topological features. We also developed a computational approach to identify candidate risk miRNA-gene-drug triplets (MGDTs) based on mRNA and miRNA expression data. The candidate risk MGDTs hold specific and strong activity score profiles in cardiac hypertrophy and AMI. We also extracted an insulin-like growth factor 1 receptor-related subnetwork in cardiac hypertrophy and a vascular endothelial growth factor A-related subnetwork in AMI. Finally, we utilized pathways to explore the mechanisms underlying cardiac hypertrophy and AMI and to identify candidate drugs for these diseases. These results provide novel insights into the underlying mechanisms and treatment of cardiac hypertrophy and AMI.

## Results

### Global properties of the MGDT network in heart disease

**Fig. 1:**
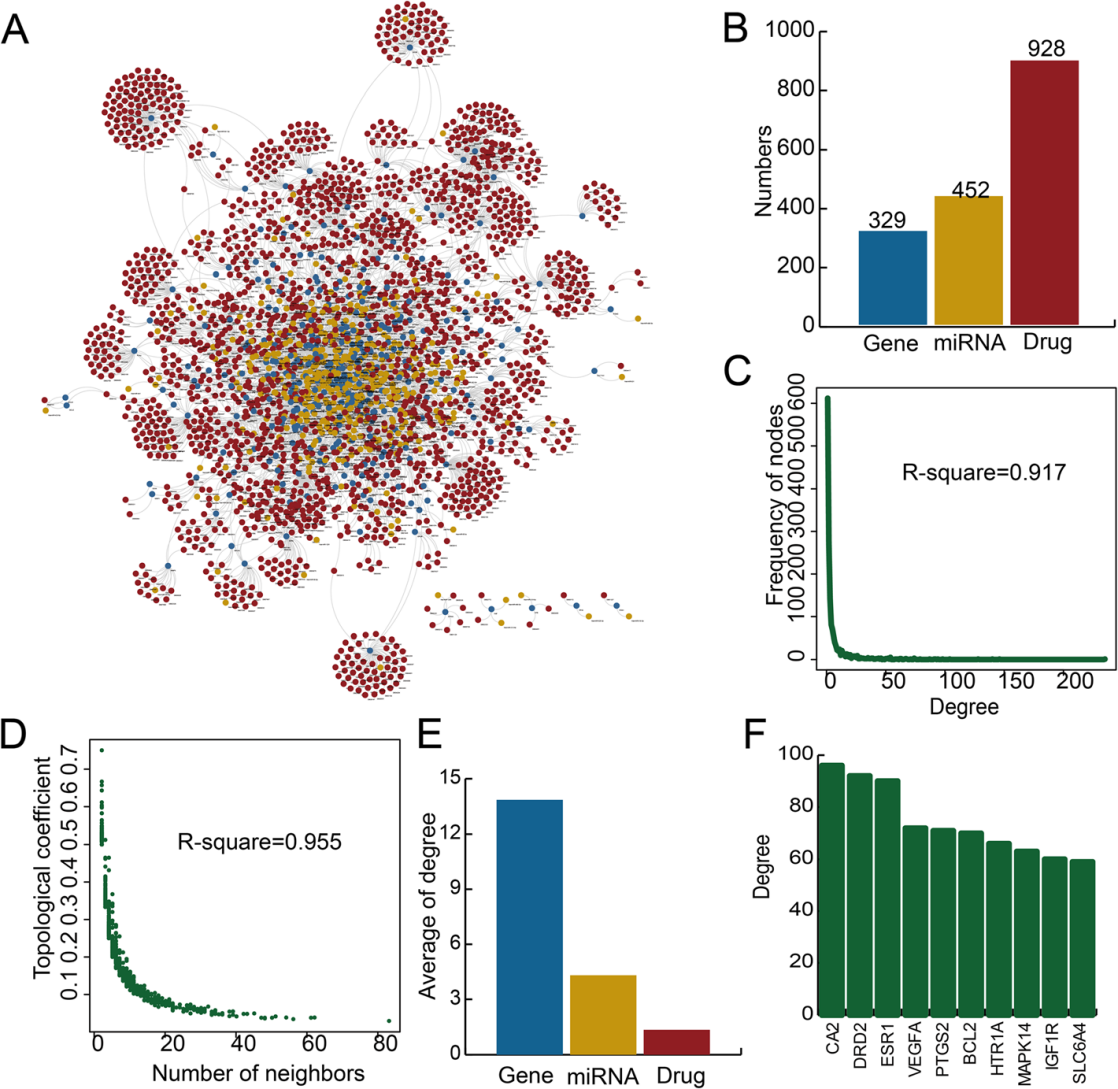
Construction and global characteristics of the MGDT networks for cardiac hypertrophy and AMI. **a** A global MGDT network for cardiac hypertrophy and AMI. Blue, yellow, and red represent coding genes, miRNAs, and drugs, respectively. **b** A bar plot showing the numbers of coding genes, miRNAs, and drugs. **c** Degree distribution of all nodes in the MGDT network. **d** Topological coefficient of all nodes in the MGDT network. **e** A bar plot showing the average degrees of coding genes, miRNAs, and drugs. **f** The degrees of nodes with the highest degrees in the whole network

We used cardiovascular disease-associated genes and experimentally verified interactions to construct a MGDT network (Fig. 1a). The MGDT network consisted of 2709 nodes (329 genes, 452 miRNAs, and 928 drugs) and 4571 edges (Fig. 1b). We found that the whole MGDT network showed a scale-free distribution (*R*^2^ = 0.917) that was close to that of the majority of biological networks (Fig. 1c). In addition, this result suggested that this MGDT network was similar to a small-world network [18]. Moreover, we found that the degree increased when the topological coefficient decreased, which indicated that the MGDT network showed a hierarchical modularity phenomenon (Fig. 1d). Other types of regulatory networks, such as the ceRNA network in cancer, showed similar topological characteristics [19, 20]. We found that miRNAs in the MGDT network held a higher degree than genes and drugs. The average degrees of the genes, miRNAs, and drugs were 13.85, 4.32, and 1.36, respectively (Fig. 1e). These results indicated that although the miRNAs did not encode proteins, they exhibited more specific topological properties than the mRNAs in the MGDT network. The highest degree nodes were CA2, DRD2, and ESR1 (Fig. 1f). These high degree nodes all play essential roles in biological processes. The above results indicated that the MGDT network showed special features of a regulatory network and might provide an effective background for drug repurposing for cardiac hypertrophy and AMI.

### Some MGDTs are specific for cardiac hypertrophy and AMI

**Fig. 2:**
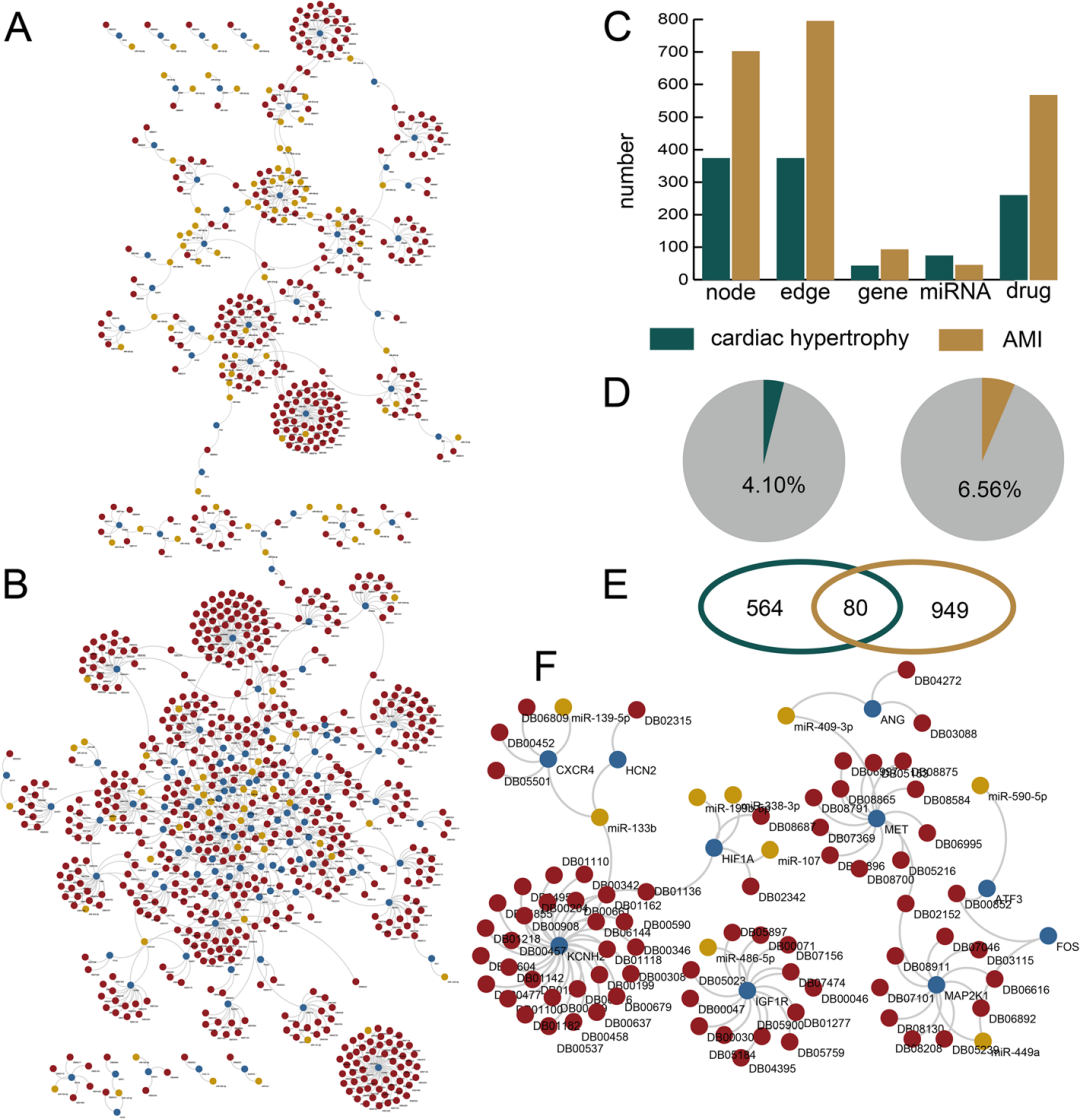
The significantly dysregulated MGDTs in the cardiac hypertrophy and AMI networks. **a** The significantly dysregulated MGDTs in the cardiac hypertrophy network. **b** The significantly dysregulated MGDTs in the AMI network. **c** The numbers of nodes, edges, genes, miRNAs, and drugs in the cardiac hypertrophy and AMI networks. **d** Pie charts showing the percentages of significantly dysregulated MGDTs out of all MGDTs in the cardiac hypertrophy and AMI networks. **e** Venn diagram showing the common dysregulated MGDTs between the cardiac hypertrophy and AMI networks. **f** The common dysregulated MGDTs in the networks

We found 15,696 MGDTs in the whole network. We identified 644 and 1029 significantly dysregulated MGDTs in cardiac hypertrophy and AMI, respectively. Then, we constructed cardiac hypertrophy- and AMI-specific networks (Fig. 2a, b). The cardiac hypertrophy-specific network consisted of 374 nodes, 373 edges, 42 genes, 73 miRNAs, and 259 drugs. The AMI-specific network consisted of 701 nodes, 794 edges, 92 genes, 44 miRNAs, and 566 drugs (Fig. 2c). A total of 4.01% of the MGDTs were significantly dysregulated in cardiac hypertrophy, and 6.56% of the MGDTs were significantly dysregulated in AMI (Fig. 2d). We also discovered 80 common MGDTs between cardiac hypertrophy and AMI (Fig. 2e). In total, we found 564 cardiac hypertrophy-specific MGDTs and 949 AMI-specific MGDTs. Then, we constructed a common MGDT network to explore the mechanisms underlying cardiac hypertrophy and AMI (Fig. 2f). The common MGDT network consisted of 91 nodes and 88 edges.

### Activity profiles of MGDTs for cardiac hypertrophy and AMI

**Fig. 3:**
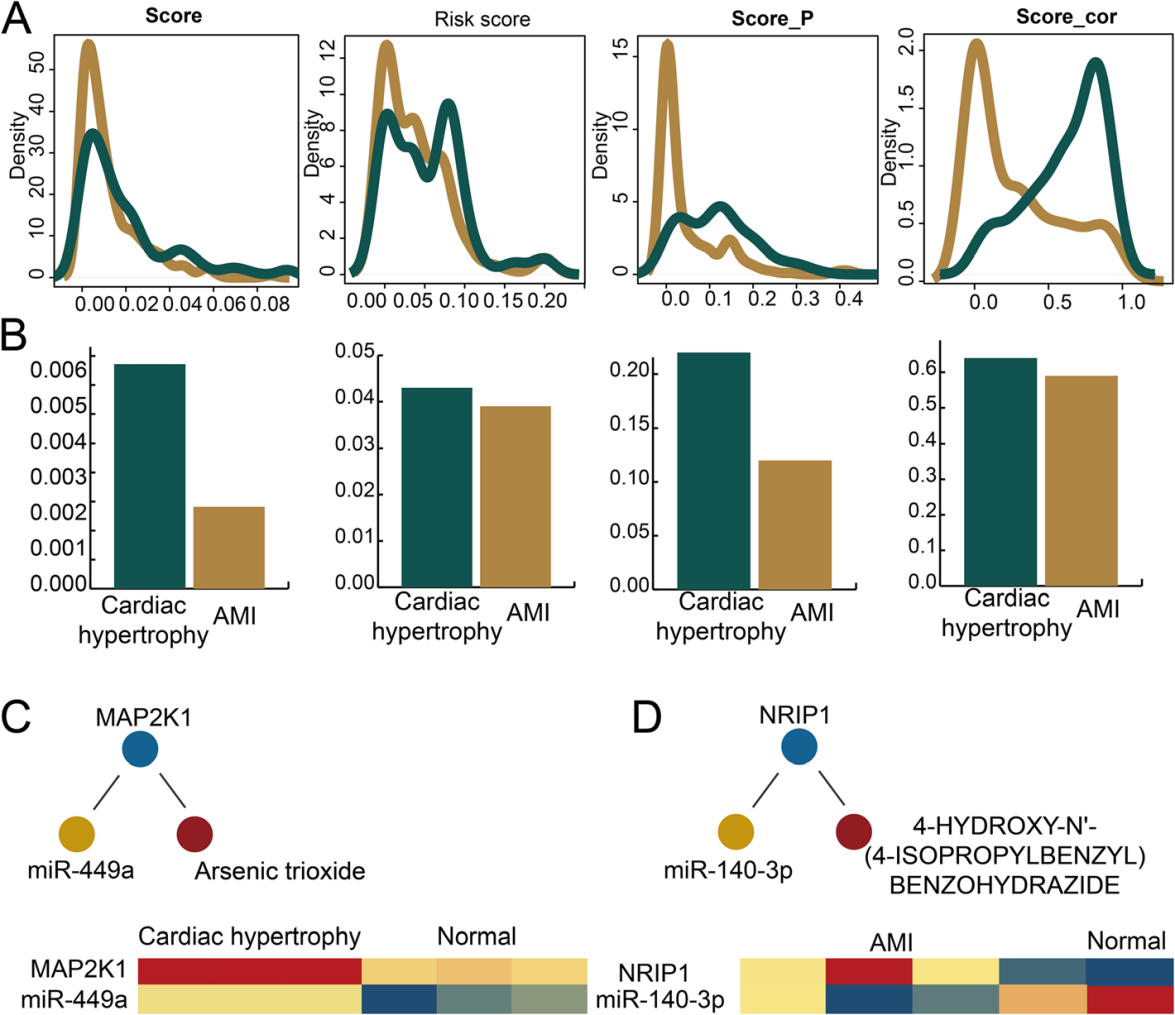
Construction of an activity score profile for MGDTs in the cardiac hypertrophy and AMI networks. **a** The density distribution curves of the last score, risk score, score_p, and score_cor. **b** Bar plots showing the four types of scores in the cardiac hypertrophy and AMI networks. **c**, **d** The top-ranked MGDTs with the highest scores in the cardiac hypertrophy and AMI networks. The heat map shows miRNA and mRNA expression

To characterize the compactness of MGDTs for cardiac hypertrophy and AMI, we evaluated the score values of all MGDTs. We evaluated four types of scores (the last score, risk score, score_p, and score_cor) for cardiac hypertrophy and AMI. We found that the density curves for these four types of scores were not all similar (Fig. 3a). For example, most last score values of these dysregulated MGDTs were concentrated between 0.01 and 0.015, and the results indicated that these dysregulated MGDTs all had strong dysregulated features. In addition, we discovered some distinctions for score_p and score_cor between cardiac hypertrophy and AMI (Fig. 3b). Although the MGDTs for cardiac hypertrophy had stronger score_cor values than those of AMI, the MGDTs for cardiac hypertrophy also had weaker score_p values. For cardiac hypertrophy, the highest MGDT score was obtained for miR-449a/MAP2K1/arsenic trioxide. miR-449a and MAP2K1 were both significantly differentially expressed (*P* = 0.001 and 0.039, respectively) (Fig. 3c). For AMI, the highest MGDT score was obtained for miR-140-3p/NRIP1/4-HYDROXY-N’-BENZOHYDRAZIDE. miR-140-3p and NRIP1 were both significantly differentially expressed (*P* = 0.09 and <  0.001, respectively) (Fig. 3d). These results indicated that dysregulated MGDTs in cardiac hypertrophy and AMI all had strong dysregulated levels.

### Functional analyses and strong MGDTs in cardiac hypertrophy and AMI

**Fig. 4:**
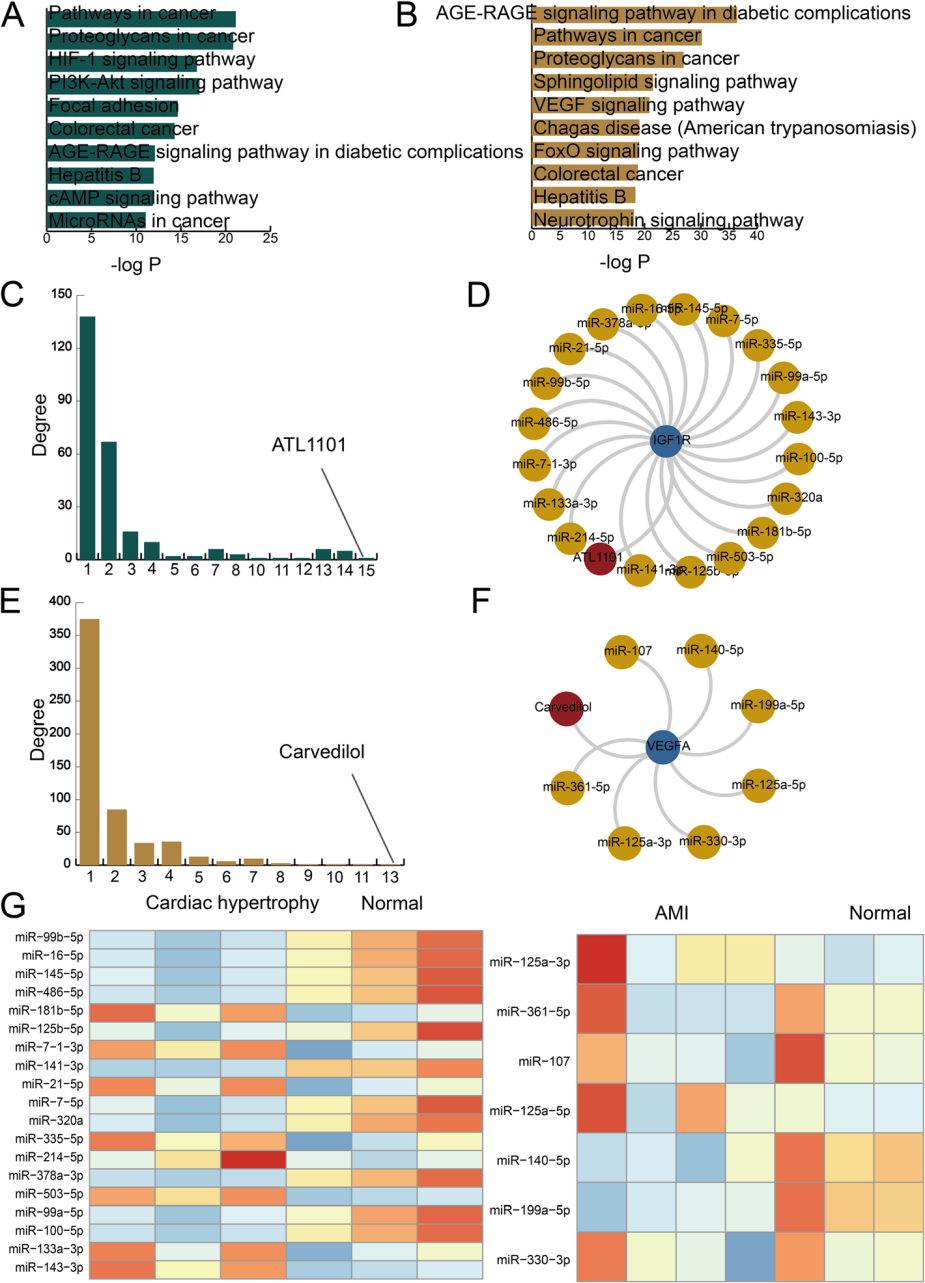
Functional analyses and construction of a miRNA-mediated subnetwork. **a** The KEGG pathway enrichment results for all genes in the dysregulated MGDTs in the cardiac hypertrophy network ranked by − log10(*P*) are presented as bar plots. **b** The KEGG pathway enrichment results for all genes in the dysregulated MGDTs in the AMI network ranked by − log10(*P*) are presented as bar plots. **c** A bar plot showing the degrees of drugs in the cardiac hypertrophy network. **d** The IGFR1-centered subnetwork of the cardiac hypertrophy network. **e** A bar plot showing the degrees of drugs in the AMI network. **f** The VEGFA-centered subnetwork in the AMI network. **g** The heat maps show miRNA expression in the two subnetworks

To explore the underlying mechanisms and identify drug repurposing candidates for cardiac hypertrophy and AMI, we performed functional analyses for all genes in the two significant dysregulated networks. We discovered that the genes in the cardiac hypertrophy and AMI networks were all enriched in some basic biological processes (Fig. 4a, b). Specifically, the genes in the cardiac hypertrophy network were enriched in the HIF-1 signaling pathway. P53-induced inhibition of HIF-1 can cause cardiac dysfunction during pressure overload [21]. The genes in the AMI network were enriched in the FOXO signaling pathway. FOXO transcription factors regulate cardiomyocyte proliferation and myocardial growth during development [22]. In addition, the genes in the cardiac hypertrophy and AMI network were enriched in a pathway named the AGE-RAGE signaling pathway, which is involved in diabetic complications. This pathway is a well-studied cascade in many different disease states, particularly diabetes. We also explored candidate drugs in the two MGDT networks. We extracted two candidate drugs that participated in most dysregulated MGDTs in cardiac hypertrophy and AMI. In cardiac hypertrophy, the drug that participated in most of the dysregulated MGDTs was ATL1101, which is a second-generation antisense drug designed to block the synthesis of the IGF-1 receptor (IGF1R), a protein involved in the regulation of cell overgrowth in psoriasis. (Fig. 4c). IGF1R plays an important role in the growth and continues to anabolic effects in adults; thus, it can induce hypertrophy of skeletal muscle and other target tissues. We also constructed an IGF1R-centered subnetwork (Fig. 4d). The network consisted of one drug, one gene, and 19 miRNAs. We found that the direct target of ATL1101 was the IGF1R gene, which was regulated by some miRNAs. We inferred that ATL1101 influenced cardiac hypertrophy via IGF1R and some essential miRNAs. In AMI, the drug that participated in most of the dysregulated MGDTs was carvedilol, which is a non-selective beta blocker indicated in the treatment of mild to moderate congestive heart failure. Its direct target is VEGFA which is a dimeric glycoprotein that plays a significant role in neurons and is considered the main and dominant inducer of blood vessel growth (Fig. 4e). We also constructed a VEGFA-centered subnetwork (Fig. 4f). The network consisted of one drug, one gene, and 7 miRNAs. Most of the miRNAs in the two subnetworks were differentially expressed. The two heatmaps showed differences between the disease and control samples in the cardiac hypertrophy and AMI networks (Fig. 4g). Some of the other top drugs in the cardiac hypertrophy network were Insulin Human, Insulin Lispro, and Insulin Pork. These top drugs were all related with insulin and indicated insulin maybe play an essential role in cardiac hypertrophy. In the AMI network, some of the other top drugs were minocycline, vandetanib, and acetylsalicylic acid.

### Identification of novel drug repurposing candidates for cardiac hypertrophy and AMI based on the miRNA-regulated drug pathway

**Fig. 5:**
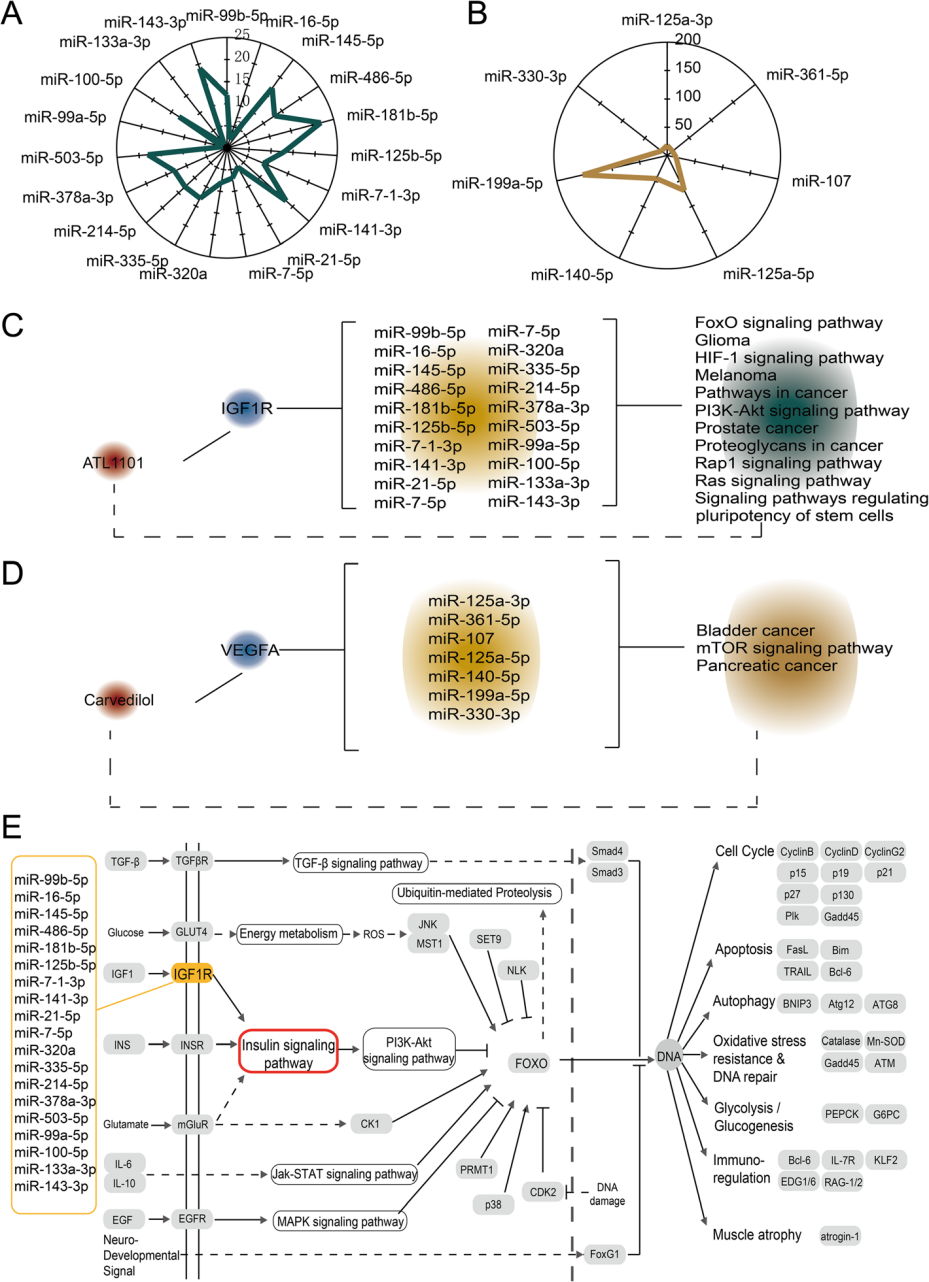
Drug repurposing candidates for cardiac hypertrophy and AMI based on miRNA-regulated drug-pathway. **a**, **b** The radar diagrams show the numbers of target genes for each miRNA in the two subnetworks. **c** The miRNA-regulated drug-pathway relationship in the cardiac hypertrophy network. **d** The miRNA-regulated drug-pathway relationship in the AMI network. **e** The FOXO signaling pathway is shown

To obtain novel drug repurposing candidates for cardiac hypertrophy and AMI, we identified miRNA-associated pathways based on the miRNA target genes. We used the target genes of each miRNA in the subnetwork to identify miRNA-associated pathways. We discovered different multiple target genes for each miRNA in the two subnetworks. miR-181b-5p had the most target genes for the IGF1R-centered subnetwork for cardiac hypertrophy (Fig. 5a). miR-199a-5p had the most target genes for the VEGFA-centered subnetwork for AMI (Fig. 5b). We also constructed a miRNA-mediated pathway and drug network. In the cardiac hypertrophy network, IGF1R was a target of ATL1101 and 19 other miRNAs (Fig. 5c). We used all target genes of these miRNAs to perform pathway enrichment. We extracted some common enrichment pathways for these miRNAs, including the FOXO and Rap1 signaling pathways. A similar regulatory relationship was also constructed for AMI (Fig. 5d). VEGFA was a target of drug carvedilol and 7 miRNAs. Pathway enrichment was also performed for all of the target genes of these miRNAs. Some common enrichment pathways, such as the mTOR signaling pathway, were extracted. We considered that these two candidate drugs were associated with cardiac hypertrophy and AMI because these common pathways were related to biological processes involved in heart disease. For example, the FOXO signaling pathway was a common enrichment pathway for cardiac hypertrophy in our analyses. We discovered that IGF1R was an upstream gene of the FOXO signaling pathway. FOXO is a key gene in the FOXO signaling pathway, and inactivation of the FOXO protein governs cell growth in the heart and thus contributes to cardiac hypertrophy [23]. Additionally, we found that the insulin signaling pathway was an essential part of the FOXO signaling pathway. Insulin-like growth factor 1 receptor can induce physiological heart growth via the phosphoinositide 3-kinase (p110a) pathway [24]. Insulin-like growth factor 1 receptor in muscle is critical for glucose homeostasis, and signaling from these receptors, particularly the insulin receptor, is required for normal cardiac metabolism and function [25]. Our results suggested that ATL1101 could be a candidate drug for cardiac hypertrophy based on the computational drug repurposing method.

## Discussion

Cardiac hypertrophy and AMI are two common heart diseases worldwide. However, cardiac hypertrophy often carries a poor prognosis due to an increased risk of arrhythmia [26] and the development of congestive heart failure. Research into the underlying mechanisms and the development of novel drugs for cardiac hypertrophy and AMI are essential. Drug repurposing is an effective, time-saving, and cost-saving method to study novel drugs for heart disease. Here, we developed a comprehensive and computational approach to perform drug repurposing for cardiac hypertrophy and AMI by integrating heart disease-associated genes, experimentally verified miRNA-gene interactions, drug-gene interactions, mRNA and miRNA expression profiles, and pathway annotation data. A global MGDT network was constructed and characterized, and cardiac hypertrophy- and AMI-specific MGDTs were identified. These specific MGDTs were important candidates for drug repurposing analyses. This comprehensive computational approach identified some significantly dysregulated MGDTs, and a KEGG pathway enrichment was performed by the target genes of miRNAs. Some candidate drugs were considered had common and unknown mechanisms of action by shared common targets of miRNA and pathways. Thus, the comprehensive computational approach could realize drug repurposing.

In our analysis, we discovered that ATL1101 was an effective drug candidate for cardiac hypertrophy. ATL1101 is an antisense compound targeting insulin-like growth factor 1 receptor, or IGF1R. Karina Huynh et al. suggested that overexpression of insulin-like growth factor 1 receptor prevented diabetes-induced cardiac fibrosis and diastolic dysfunction. Knezevic et al. demonstrated the existence of a negative feedback loop between miR-378, IGF1R, and IGF1R that was associated with postnatal cardiac remodeling and regulation of cardiomyocyte survival during stress [27]. Here, we suggested that IGF1R influenced cardiac hypertrophy through the FOXO signaling pathway by interacting with a series of miRNAs. In addition, other candidate drugs, such as Insulin Human, Insulin Lispro, and Insulin Pork which were all investigational or approved insulin-related drugs, were identified in our analysis as drug repurposing candidates for cardiac hypertrophy. The computational approach for drug repurposing provided novel insights into mechanistic and drug candidate research in cardiac hypertrophy and AMI.

miRNAs regulate gene expression through translational repression and degradation of mRNAs and significantly contribute to post-transcriptional regulation of drugs [28]. Downregulation of miR-455-3p could be linked to proliferation and drug resistance of pancreatic cancer cells via targeting TAZ [29]. Lopez-Riera et al. indicated that steatotic drugs induced a common set of hepatic miRNAs that could be used in drug screening during preclinical development for fatty liver disease [30]. In our analyses, we also considered the essential role of miRNAs in drug repurposing. We constructed miRNA-mediated drug and pathway regulatory relationship networks for cardiac hypertrophy and AMI and identified enriched pathways based on miRNA target genes. Our results suggested that integrating miRNA interactions and expression was essential for drug repurposing. Well-designed experiments or clinical trials should be conducted in future works to determine whether these repurposing candidate drugs were applicable for the treatment of cardiac hypertrophy and AMI.

## Conclusions

In summary, we constructed a miRNA-gene-drug network and analyzed its topological features based on heart disease-associated genes and experimentally verified gene-miRNA and gene-drug interactions. We also presented a computational and integrated approach to identify candidate risk MGDTs, followed by mRNA and miRNA expression profiles, in cardiac hypertrophy and AMI. The candidate risk MGDTs had specific and strong activity score profiles in the cardiac hypertrophy and AMI networks. The IGFR1-related subnetwork in cardiac hypertrophy and the VEGFA-related subnetwork in AMI were extracted for analysis. Finally, we explored the underlying mechanisms and identified candidate drugs for cardiac hypertrophy and AMI based on common pathways of target genes for all miRNAs in the subnetworks. Collectively, our results contributed to a better understanding of the relationships among genes, miRNAs, pathways, and drugs and identified promising drug repurposing candidates for cardiac hypertrophy and AMI.

## Methods

### Collection of high-throughput miRNA and gene data

We downloaded expression profiles for cardiac hypertrophy and AMI from the Gene Expression Omnibus database (www.ncbi.nlm.nih.gov/geo). The two relevant studies included both miRNA and gene expression profiles from the same sample. A total of three disease samples and three control samples for cardiac hypertrophy were obtained from GSE60291 [31], and four disease samples and one control sample for AMI were obtained from GSE24591 (unpublished data).

### Cardiovascular disease-associated genes

We downloaded cardiovascular disease-associated genes from DisGeNET, which integrates disease-associated genes and variants from expert-curated repositories, GWAS catalogs, animal models, and the scientific literature [32]. We extracted keywords for diseases named “heart” or “cardiac”. Finally, we found 2293 cardiovascular disease-associated genes and their corresponding risk scores for subsequent analysis.

### Experimentally validated miRNA-gene and gene-drug interactions

We downloaded the gene-miRNA associations from the public database miRTarBase 7.0, which accumulated more than three hundred and sixty thousand miRNA-gene interactions [33]. We only extracted experimentally validated miRNA-gene interactions. Then, we obtained gene-drug interaction data from DrugBank, which is a web-enabled database containing comprehensive molecular information about drugs, their mechanisms, their interactions, and their targets [34]. We constructed a MGDT network based on these experimentally validated interactions used Cytoscape 3.0 (http://www.cytoscape.org/).

### Dissecting topological characteristics of the MGDT network

We used the degree distributions and topological coefficients to assess the entire MGDT network for all nodes. All analyses were performed using Cytoscape 3.0 (http://www.cytoscape.org/).

### Identification of cardiac hypertrophy- and AMI-specific MGDTs based on expression data

We designed an integrative and computational approach to identify specific MGDTs in cardiac hypertrophy and AMI that used the MGDT regulatory networks and the miRNA and mRNA expression data. First, we used Student’s *t* test to compare differences in mRNA and miRNA expression between patients with disease (cardiac hypertrophy and AMI) and the corresponding controls for each MGDT. Second, we calculated Pearson’s correlation coefficients (PCCs) for mRNA and miRNA interactions for each MGDT in the disease and control samples. We calculated the significant difference between the disease and control samples. The absolute difference in the PCCs between the disease and control samples was obtained for the subsequent analysis. We presented three comprehensive scores (*S*_risk,_ *S*_P,_ and *S*_PCC_) to evaluate the dysregulated levels for each MGDT in cardiac hypertrophy and AMI. The equations were as follows:

- *S*_risk_ = risk − score
- *S*_*P*_ = *P*_mRNA_^∗^*P*_miRNA_
- *S*_pcc_ =  ∣ *D*_pcc_ − *C*_pcc_∣

In the three equations, *S*_risk_ represents the verified level between the disease and genes. *P*_mRNA_ and *P*_miRNA_ represent the *P* values of mRNAs and miRNAs, respectively, for each MGDT derived from the above *t* test. *S*_P_ is the difference between the expression level of the MGDT between the samples with disease and the corresponding controls. *D*_pcc_ and *C*_pcc_ refer to the PCCs of the mRNA-miRNA interaction pairs for the disease and control samples, respectively. *S*_pcc_ is the absolute distinction of the PCC score between the disease and control samples in each MGDT. We used an equally weighted approach to rank all MGDTs following the above three comprehensive scores [35]. We obtained three ranked lists, and the ranking positions in the three lists were used to calculate the last ranking score list for each MGDT in the cardiac hypertrophy and AMI networks. Higher ranking scores referred to stronger dysregulated MGDTs between the disease and control samples. The permutation-based final ranking scores were generated by randomly disturbing all sample labels in the expression profiles 1000 times. We compared each final ranking score of a MGDT with the permutation-based final ranking score to obtain significant *P* values (*P* < 0.05). All analyses were performed using the R software (version 3.2.3; https://www.r-project.org/).

### Pathway enrichment analysis

We used the Database for Annotation, Visualization, and Integrated Discovery (DAVID) to annotate enriched pathways for the genes [36]. We extracted enriched pathways using the Kyoto Encyclopedia of Genes and Genomes (KEGG) with a *P* value cutoff of 0.05. Then, we used the target gene sets of miRNAs to identify miRNA-associated pathways. Finally, we constructed relationships, including drugs, pathways, genes, and miRNAs.

## Abbreviations

AMI: Acute myocardial infarction
GEO: Gene Expression Omnibus
KEGG: Kyoto Encyclopedia of Genes and Genomes
MGDTs: miRNA-gene-drug triplets
miRNAs: microRNAs
PCCs: Pearson correlation coefficients
